# Prognostic role of pretreatment thrombocytosis on survival in patients with cervical cancer: a systematic review and meta-analysis

**DOI:** 10.1186/s12957-019-1676-7

**Published:** 2019-08-02

**Authors:** Weijuan Cao, Xiaomin Yao, Danwei Cen, Yajun Zhi, Ningwei Zhu, Liyong Xu

**Affiliations:** 1 College of Pharmacy, Zhejiang Pharmaceutical College, Ningbo, 315100 Zhejiang Province China; 2 Zhejiang Pharmaceutical College, No. 888, East Section of Yinxian Avenue, Higher Education Park, Ningbo, 315100 Zhejiang Province China

**Keywords:** Meta-analysis, Prognosis, Thrombocythemia, Cervical cancer

## Abstract

**Background:**

This meta-analysis summarized the prognostic role of an elevated platelet count before treatment on survival outcomes in patients with cervical cancer.

**Methods:**

The PubMed, Embase, and Cochrane library electronic databases were systematically searched for studies reporting the effect estimates with 95% confidence intervals (CIs) of pretreatment thrombocytosis on survival from the database inceptions to December 2018. The pooled hazard ratios (HRs) with 95% CIs for overall survival (OS), progression-free survival (PFS), and recurrence-free survival (RFS) were calculated using random-effects models.

**Results:**

Nineteen retrospective studies that recruited 6521 patients with cervical cancer were eligible for this study. The summary results indicated that an elevated platelet count was significantly associated with a poor OS (HR 1.50; 95% CI 1.19–1.88; *P* = 0.001), PFS (HR 1.33; 95% CI 1.07–1.64; *P* = 0.010), and RFS (HR 1.66; 95% CI 1.20–2.28; *P* = 0.002). Sensitivity analysis indicated that the pooled PFS was variable after sequential exclusion of individual studies. The predictive value of pretreatment thrombocytosis on OS differed according to the publication year (*P* = 0.039), country (*P* = 0.013), and sample size (*P* = 0.029), and the role of pretreatment thrombocytosis on PFS could be affected by the study quality (*P* = 0.046).

**Conclusion:**

The findings of this study indicated that an elevated platelet count before treatment was associated with poor OS, PFS, and RFS. These results require further verification in large-scale prospective studies.

**Electronic supplementary material:**

The online version of this article (10.1186/s12957-019-1676-7) contains supplementary material, which is available to authorized users.

## Background

Cervical cancer has the second highest incidence and the fourth leading mortality due to cancer in women worldwide, with a reported 527,000 new cases and 265,700 deaths annually [1]. Nearly 85% of cervical cancer cases occur in developing countries and women aged 40–45 years have the highest disease incidence [2]. Epidemiologic studies have identified several factors that could affect the progression of cervical cancer, including human papillomavirus, oral contraceptives, sexual promiscuity, and smoking [3–6]. Currently, radical hysterectomy with pelvic lymph node dissection is widely used for the treatment of early-stage cervical cancer, although recurrences occur in nearly 25% of patients [7, 8]. Concurrent chemoradiotherapy is the standard treatment strategy in patients with invasive cervical cancer, with a risk of recurrence ranging from 10 to 20% in patients with stage Ib to IIa disease and 50 to 70% in patients with stage IIb to IVa disease [9]. Therefore, effective prognostic factors should be explored to predict survival outcomes in patients with cervical cancer.

The incidence of thrombocytosis ranged from 4 to 55% of patients with malignant tumors at initial diagnosis and during the course of the disease, which may be due to various cytokines and growth factors [10, 11]. Inflammatory responses caused by cancer might play an important role in tumor development including cancer initiation, promotion, malignancy conversion, invasion, and metastasis at various stages [12]. Numerous studies have demonstrated the prognostic role of inflammatory biomarkers on survival in patients with various diseases, including platelet count, anemia, and red cell distribution width [13–15]. Moreover, previous studies have indicated that tumor-derived interleukin-6 could stimulate thrombopoiesis, leading to thrombocytosis and tumor progression in patients with ovarian cancer [16]. However, the prognostic role of platelet count in patients with cervical cancer remains controversial. As the measurement of platelet count is economical and easily accessible in clinical practice, we conducted a systematic review and meta-analysis to verify the prognostic value of thrombocytosis on survival outcomes in patients with cervical cancer to identify an additional effective biomarker.

## Methods

### Data sources, search strategy, and selection criteria

The current meta-analysis was conducted and reported following the Preferred Reporting Items for Systematic Reviews and Meta-Analysis Statement issued in 2009 [17]. We searched PubMed, Embase, and the Cochrane library for studies that investigated the prognostic role of thrombocytosis on survival outcomes in patients with cervical cancer from the inception of the databases up to December 2018 using the following search terms as medical subject headings and free words: (“thrombocytosis” or “thrombocythemia” or “platelet count” or “platelet”) AND (“cervical cancer” or “cervical tumor” or “cervical neoplasm” or “cervical carcinoma”) AND (“prognosis” or “outcome” or “survival” or “mortality” or “recurrence” or “progression” or “metastasis”). After the selection of potentially eligible studies based on the inclusion criteria, manual searches of the reference lists of the retrieved studies were also conducted to identify additional studies for consideration.

The literature search and study selection were conducted independently by two authors and a third author made the final decision if cases of disagreement. A study was included if it met the following inclusion criteria: (1) study design: both prospective or retrospective studies were included; (2) patients: patients in retrieved studies diagnosed with cervical cancer, irrespective of disease stages; (3) exposure: platelet count or thrombocytosis were measured before treatment; (4) control: the platelet count before treatment was normal in the control group; and (5) outcomes: the study should report at least one of following outcomes: overall survival (OS), progression-free survival (PFS), and recurrence-free survival (RFS). Study designed as review, reported other hematological markers and other outcomes were excluded.

### Data collection and quality assessment

The collected data included first authors’ surname, publication year, country, study design, sample size, mean age, disease stages, treatment strategy, platelet count cutoff, adjusted factors, and reported outcomes. Study quality was evaluated using the Newcastle-Ottawa Scale (NOS), which is the most commonly used tool for evaluating the quality of observational studies in meta-analyses [18]. The NOS system is based on selection (4 items), comparability (1 item), and outcome (3 items), with a star system ranging from 0 to 9 for quality assessment. The data collection and quality assessment were carried out by two authors, with inconsistencies resolved by an additional author referring to the original article.

### Statistical analysis

The pooled hazard ratios (HRs) with 95% confidence intervals (CIs) for OS, PFS, and RFS were calculated using the adjusted or crude HRs and 95% CIs reported in individual studies. All pooled results were calculated using a random-effects model, allowing for the true underlying effect to vary among included studies [19, 20]. The heterogeneity across the included studies was assessed as proposed by Higgins, which provides the percentage of total variation among included studies [21]. Moreover, *P* values for *Q* statistics were calculated, with *P* < 0.100 indicating significant heterogeneity [22]. Sensitivity analysis was conducted for OS, PFS, and RFS to evaluate the impacts of single studies on the overall analysis [23]. Subgroup analyses were also performed based on publication year, country, sample size, mean age, treatment strategy, cutoff value, adjusted or not, and study quality. *P* values between subgroups were calculated using chi-square tests to explore the difference of the effect estimates between subgroups [24]. Publication biases for OS, PFS, and RFS were calculated using funnel plots, Egger [25], and Begg [26] test results. The *P* values for all pooled results were two-sided, and *P* < 0.05 was considered statistically significant. All analyses were conducted using STATA (version 10.0; Stata Corporation, College Station, TX, USA).

## Results

### Literature search

**Fig. 1:**
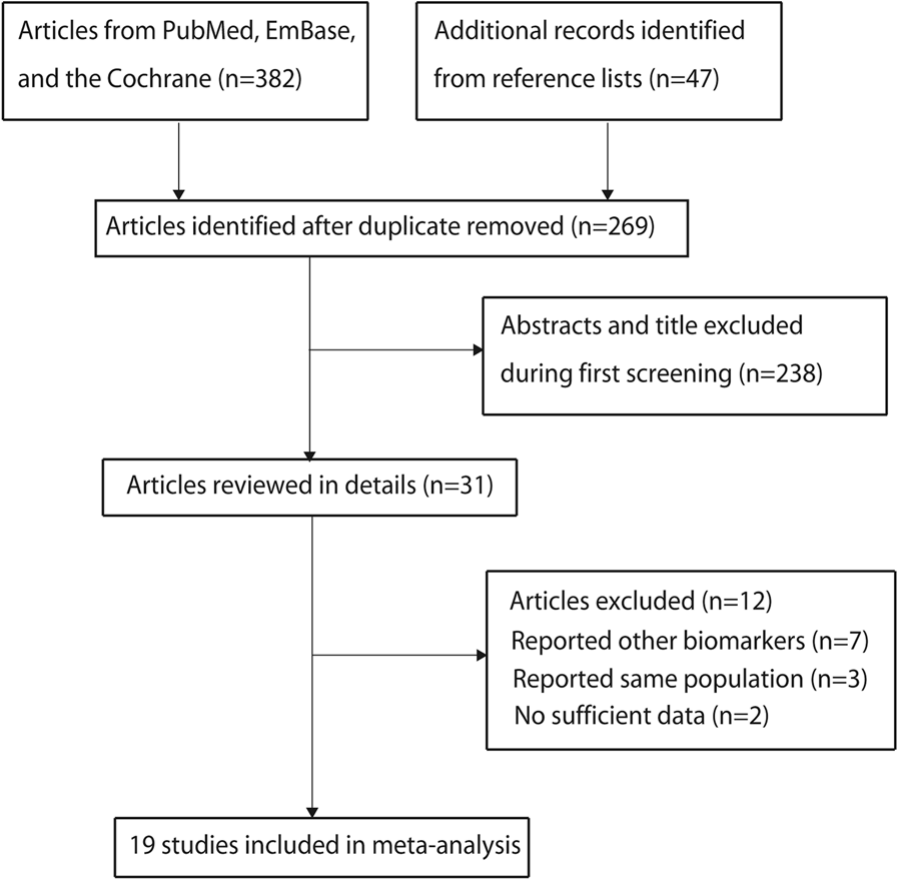
Flow diagram of the literature search and study selection process

A total of 382 records were identified in the initial search of the PubMed, EmBase, and Cochrane library electronic databases; of these, 160 duplicated and 238 irrelevant records were excluded. The remaining 31 studies were retrieved for full-text evaluations, and 12 studies were excluded for the following: reported other biomarkers (*n* = 7), reported on the same population (*n* = 3), and insufficient data (*n* = 2). No new eligible studies were obtained in the manual searches of the reference lists of the remaining studies. Finally, a total of 19 studies were included in the present study [27–45]. The flow diagram of the study inclusion is presented in Fig. 1.

### Study characteristics

We identified a total of 19 retrospective studies including 6521 patients with cervical cancer. The baseline characteristics of included studies or patients are presented in Table 1. These studies were published in 1992–2018, and the sample sizes ranged from 46 to 1189. The included studies were conducted in China (*n* = 7), the USA (*n* = 4), Japan (*n* = 2), Italy (*n* = 2), England (*n* = 1), South Africa (*n* = 1), Poland (*n* = 1), and Canada (*n* = 1). Ten of the studies included patients diagnosed at early stages, and the remaining nine studies included patients at all stages. The quality of the included studies is shown in the last column of Table 1. Six studies had seven stars, nine studies had six stars, and the remaining four studies had five stars.

**Table 1:** Baseline characteristics of studies included in the meta-analysis

Study	Publication year	Country	Study design	Sample size	Mean age (years)	Disease stages	Treatment strategy	Cutoff value	Adjusted or not	Reported outcomes	NOS scale
Hernandez et al. [33]	1992	USA	Retrospective	113	59.2	I–IV	Radiation	400	Not	OS	5
Lopes et al. [37]	1994	England	Retrospective	643	45.5	Ib–IV	Surgery or radiation	400	Not	OS	7
Hernandez et al. [32]	1994	USA	Retrospective	623	NS	Ib	Surgery	400	Not	OS, PFS	7
Rodriguez et al. [40]	1994	USA	Retrospective	219	40.0	Ib	Surgery	300	Not	OS	5
De Jonge et al. [28]	1999	South Africa	Retrospective	93	NS	Ib	Surgery	400	Yes	OS, RFS	6
Hernandez et al. [31]	2000	USA	Retrospective	291	49.8	IIb–IVa	Surgery or radiation	400	Yes	OS	6
Qiu et al. [39]	2010	China	Retrospective	318	43.0	I–IV	NS	400	Not	OS	6
Gadducci et al. [29]	2010	Italy	Retrospective	46	47.0	Ib–IIb	Surgery or chemotherapy	272	Not	OS, RFS	6
Gadducci et al. [30]	2010	Italy	Retrospective	140	47.0	Ib–IIb	Surgery or chemotherapy	272	Not	OS, RFS	6
Wang et al. [41]	2012	China	Retrospective	111	42.0	Ib–IIb	Surgery or chemotherapy	266	Not	OS, PFS	5
Biedka et al. [27]	2012	Poland	Retrospective	58	NS	I–IV	Surgery or radiation	NS	Not	PFS	5
Zhao et al. [44]	2015	China	Retrospective	220	NS	I–IIa	Surgery	300	Not	OS, RFS	6
Xiao et al. [42]	2015	China	Retrospective	238	52.0	I–IV	Radiation and chemotherapy	200	Not	OS, PFS	6
Li et al. [36]	2015	China	Retrospective	380	51.0	Ia–IIb	Surgery	300	Not	OS	6
Koulis et al. [34]	2017	Canada	Retrospective	257	50.0	Ib–IV	Chemoradiotherapy and surgery	400	Yes (OS), No (PFS)	OS, PFS	7
Kozasa et al. [35]	2017	Japan	Retrospective	684	NS	I–IV	Chemoradiotherapy and surgery	350	Yes	OS, PFS	7
Zheng et al. [45]	2017	China	Retrospective	800	49.5	Ia–IIa	Surgery	272	Yes	OS, RFS	7
Nakamura et al. [38]	2018	Japan	Retrospective	98	65.0	I–IV	Radiation and chemotherapy	350	Not	OS, PFS	6
Xu et al. [43]	2018	China	Retrospective	1189	NS	Ia–IIa	Surgery	300	Yes	PFS	7

### Overall survival

**Fig. 2:**
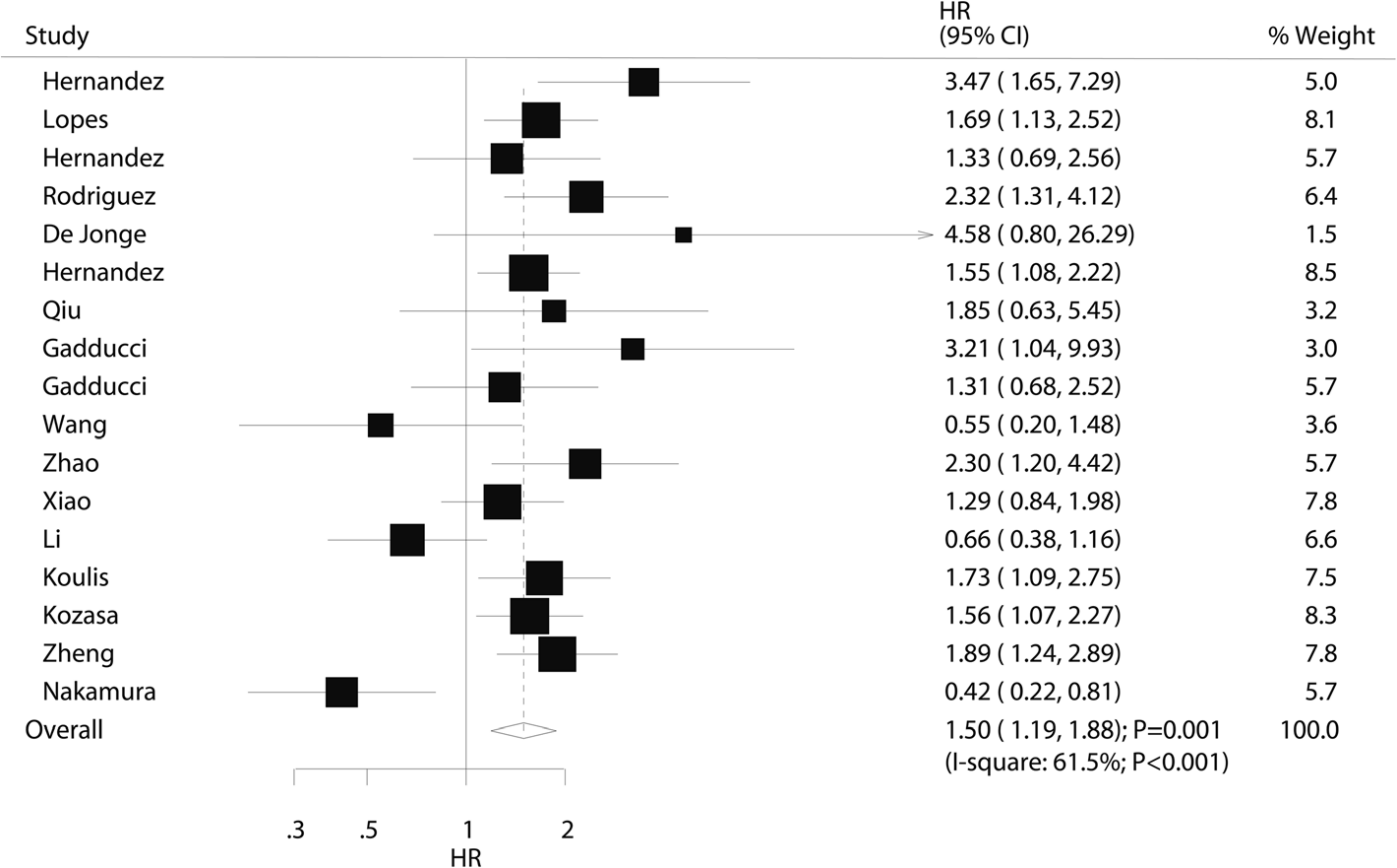
The prognostic role of pretreatment thrombocytosis on overall survival in patients with cervical cancer

The prognostic value of pretreatment thrombocytosis on OS was available in 17 studies. Overall, thrombocytosis before treatment was associated with a poor OS (HR 1.50; 95% CI 1.19–1.88; *P* = 0.001; Fig. 2). Moreover, significant heterogeneity across studies was observed (*I*^2^ 61.5%; *P* < 0.001). Sensitivity analysis revealed that the conclusion was not altered after sequential exclusion of individual studies (Additional file 1). The results of subgroup analyses indicated the results in most subsets were consistent with overall analysis, whereas pretreatment thrombocytosis did not affect the OS when pooled studies published in 2010 or after, studies conducted in Eastern countries, studies with sample size < 100, mean patients age ≥ 50.0 years, platelet cutoff value < 300, pooled crude results, and studies with lower quality (Table 2). The results of the publication bias analysis are presented in Additional file 2, and the Egger (*P* = 0.916) and Begg (*P* = 0.537) test results showed no significant publication bias for OS.

**Table 2:** Subgroup analyses for OS, PFS, and RFS

Outcomes	Factors	Groups	HR and 95% CI	*P* value	Heterogeneity (%)	*P* value for heterogeneity	*P* value between subgroups
OS	Publication year	Before 2010	1.85 (1.43–2.40)	< 0.001	22.3	0.266	0.039
		2010 or after	1.29 (0.94–1.77)	0.111	67.6	0.001	
	Country	Eastern	1.15 (0.77–1.72)	0.485	74.2	<0.001	0.013
		Western	1.78 (1.47–2.15)	<0.001	4.2	0.400	
	Sample size	≥ 100	1.56 (1.28–1.89)	<0.001	44.6	0.036	0.029
		< 100	1.64 (0.31–8.64)	0.562	85.0	0.001	
	Mean age (years)	≥ 50.0	1.16 (0.63–2.13)	0.638	83.7	<0.001	0.055
		< 50.0	1.67 (1.34–2.08)	<0.001	17.1	0.295	
		Not reported	1.68 (1.26–2.24)	<0.001	0.0	0.425	
	Treatment strategy	Surgery alone	1.64 (1.05–2.57)	0.031	66.0	0.012	0.774
		Other	1.41 (1.06–1.89)	0.020	65.9	0.002	
	Cutoff value	≥ 300	1.53 (1.14–2.05)	0.004	67.7	<0.001	0.851
		< 300	1.43 (0.97–2.11)	0.069	46.4	0.113	
	Adjusted	Yes	1.68 (1.38–2.05)	<0.001	0.0	0.748	0.149
		No	1.39 (0.98–1.96)	0.063	70.7	<0.001	
	Study quality	High	1.67 (1.37–2.03)	<0.001	0.0	0.917	0.174
		Low	1.44 (1.00–2.07)	0.052	71.6	<0.001	
PFS	Publication year	Before 2010	1.54 (0.80–2.96)	0.196	–	–	0.699
		2010 or after	1.29 (1.02–1.64)	0.036	32.4	0.170	
	Country	Eastern	1.18 (0.90–1.55)	0.241	33.7	0.183	0.115
		Western	1.71 (1.22–2.39)	0.002	0.0	0.790	
	Sample size	≥ 100	1.39 (1.16–1.67)	<0.001	0.0	0.442	0.235
		< 100	1.08 (0.22–5.24)	0.919	69.2	0.071	
	Mean age (years)	≥ 50.0	1.18 (0.72–1.94)	0.508	63.8	0.063	0.215
		< 50.0	0.66 (0.27–1.62)	0.365	–	–	
		Not reported	1.48 (1.16–1.89)	0.001	0.0	0.756	
	Treatment strategy	Surgery alone	1.31 (0.93–1.84)	0.127	0.0	0.797	0.794
		Other	1.28 (0.92–1.78)	0.144	49.9	0.076	
	Cutoff value	≥ 300	1.42 (1.12–1.80)	0.004	19.8	0.284	0.228
		< 300	1.02 (0.63–1.65)	0.936	23.6	0.253	
		Not reported	2.72 (0.61–12.10)	0.189	–	–	
	Adjusted	Yes	1.45 (1.11–1.88)	0.006	0.0	0.547	0.539
		No	1.23 (0.86–1.75)	0.266	43.9	0.113	
	Study quality	High	1.52 (1.24–1.88)	<0.001	0.0	0.789	0.046
		Low	0.96 (0.57–1.60)	0.867	37.8	0.185	
RFS	Publication year	Before 2010	8.50 (0.78–92.40)	0.079	–	–	0.173
		2010 or after	1.60 (1.22–2.10)	0.001	0.7	0.388	
	Country	Eastern	1.71 (1.24–2.34)	0.001	0.0	0.587	0.624
		Western	1.98 (0.75–5.24)	0.167	53.9	0.114	
	Sample size	≥ 100	1.55 (1.17–2.04)	0.002	0.0	0.369	0.140
		< 100	3.35 (1.25–9.00)	0.017	0.0	0.400	
	Mean age (years)	< 50.0	1.51 (1.04–2.18)	0.029	17.7	0.297	0.308
		Not reported	2.52 (0.82–7.71)	0.105	29.0	0.235	
	Treatment strategy	Surgery alone	1.75 (1.28–2.40)	<0.001	0.3	0.367	0.399
		Other	1.54 (0.64–3.73)	0.334	53.7	0.141	
	Cutoff value	≥ 300	2.52 (0.82–7.71)	0.105	29.0	0.235	0.308
		< 300	1.51 (1.04–2.18)	0.029	17.7	0.297	
	Adjusted	Yes	2.40 (0.59–9.69)	0.221	45.3	0.176	0.872
		No	1.63 (1.00–2.66)	0.048	33.8	0.221	
	Study quality	High	1.60 (1.09–2.36)	0.017	–	–	0.886
		Low	1.78 (1.04–3.05)	0.034	38.2	0.183	

### Progression-free survival

**Fig. 3:**
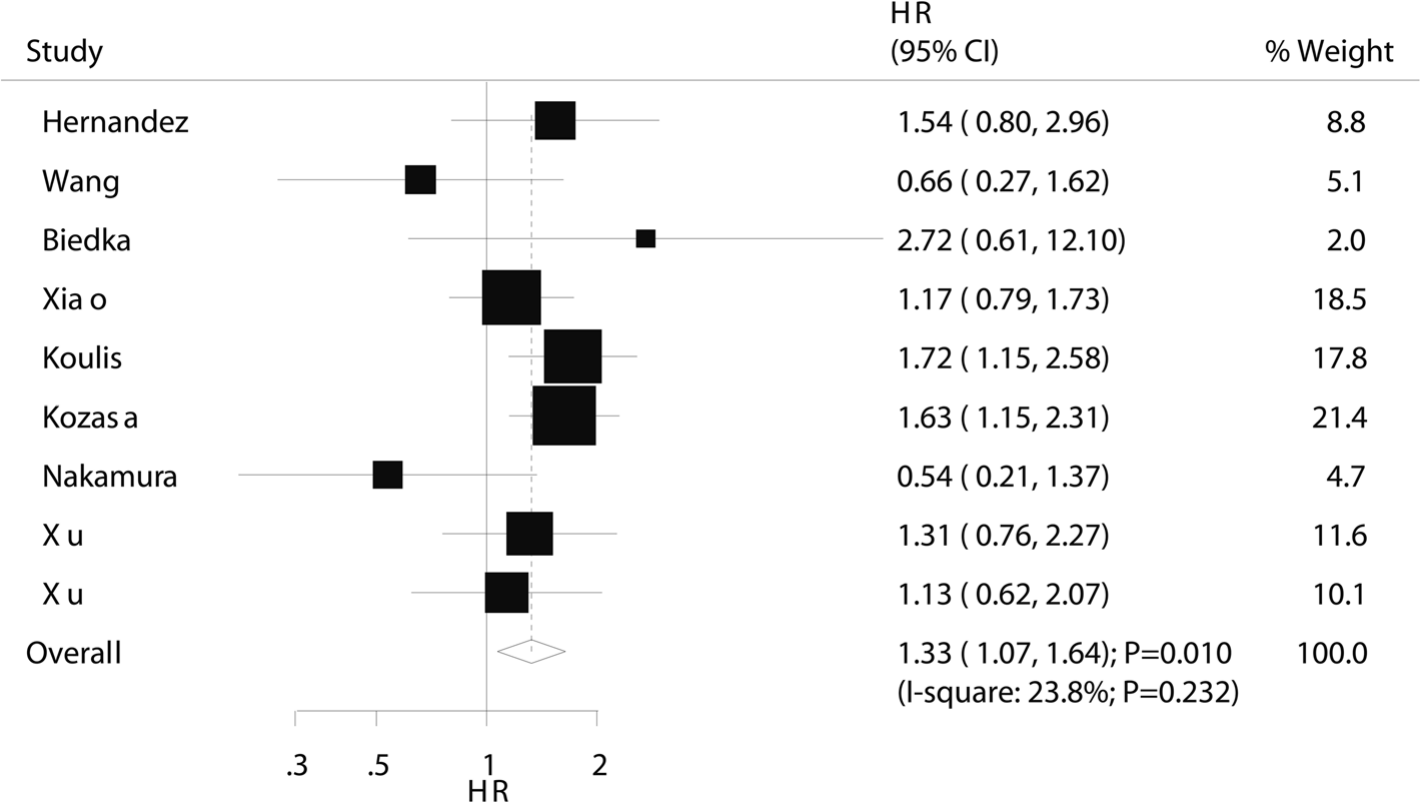
The prognostic role of pretreatment thrombocytosis on progression-free survival in patients with cervical cancer

The prognostic value of pretreatment thrombocytosis on PFS was available in eight studies. Thrombocytosis before treatment was associated with a poor PFS (HR 1.33; 95% CI 1.07–1.64; *P* = 0.010; Fig. 3), and non-significant heterogeneity was also observed (*I*^2^ 23.8%; *P* = 0.232). The pooled results varied due to marginal 95% CI values (Additional file 1). Subgroup analyses indicated that pretreatment thrombocytosis was associated with a poor PFS in studies published in 2010 or after, studies conducted in Western countries, in sample sizes ≥ 100, in studies that did not report a mean age, platelet cutoff ≥ 300, pooled adjusted results, and studies with high quality (Table 2). There was no significant publication bias for PFS (Egger and Begg *P* values 0.259 and 0.348, respectively; Additional file 2).

### Recurrence-free survival

**Fig. 4:**
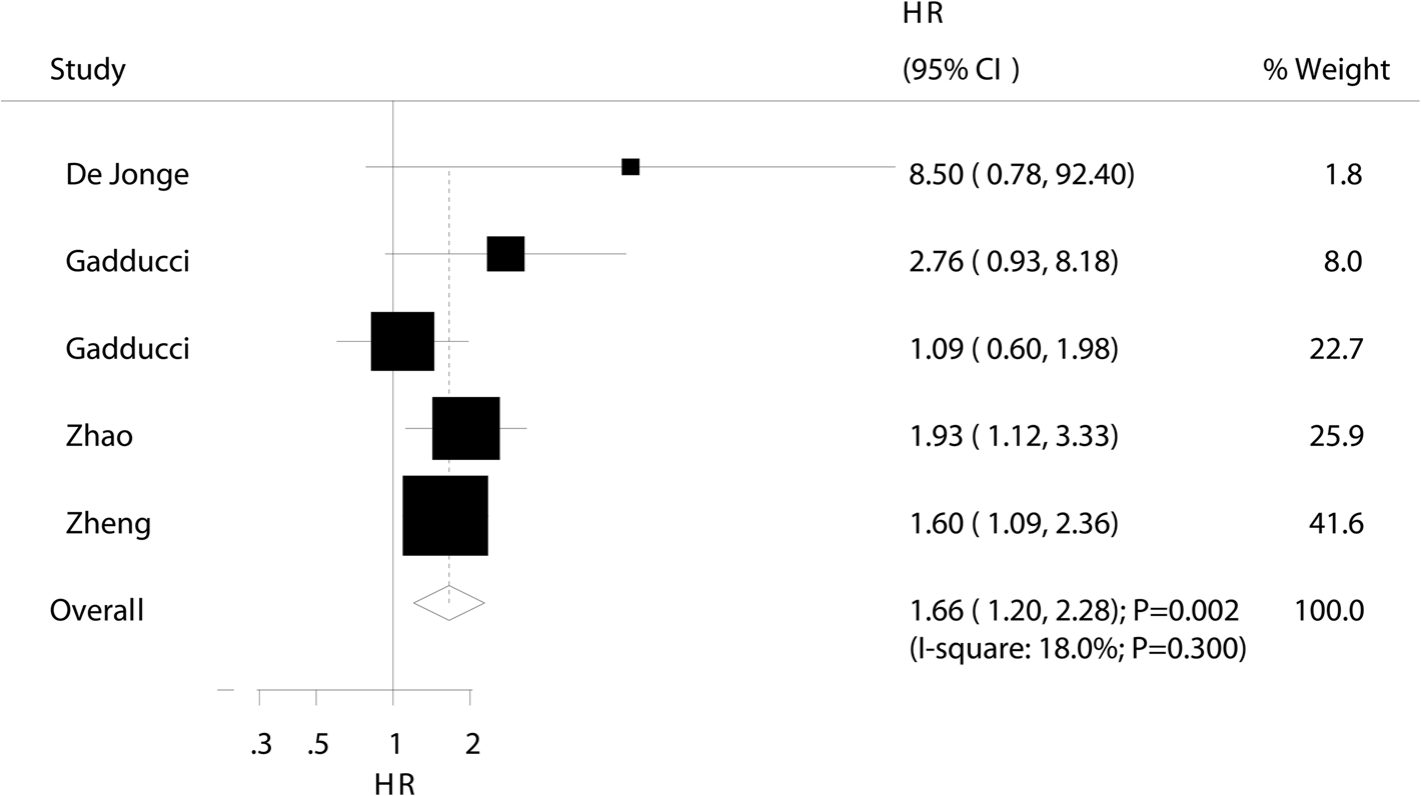
The prognostic role of pretreatment thrombocytosis on recurrence-free survival in patients with cervical cancer

The prognostic value of pretreatment thrombocytosis on RFS was available in five studies. The summary HR indicated that pretreatment thrombocytosis was associated with a poor RFS (HR 1.66; 95% CI 1.20–2.28; *P* = 0.002; Fig. 4) and nonsignificant heterogeneity was observed across the included studies (*I*^2^ 18.0%; *P* = 0.300). The results of sensitivity analysis indicated that the pooled result was stable after excluding any single study (Additional file 1). Subgroup analysis indicated that this significant association was observed mostly in subsets, whereas pretreatment thrombocytosis could not affect RFS when pooled studies published before 2010, studies conducted in Western countries, studies that did not report a mean age, in patients who received other treatment strategies, platelet cutoff ≥ 300, and pooled adjusted results (Table 2). No evidence of publication bias was observed (Egger and Begg *P* values 0.235 and 0.221, respectively; Additional file 2).

## Discussion

The current meta-analysis performed a comprehensive search for published articles and explored the prognostic role of pretreatment thrombocytosis on survival outcomes in patients with cervical cancer. This quantitative study involved 6521 patients from 19 retrospective studies with a wide range of patient characteristics. The results of this study indicated that thrombocytosis before treatment was associated with poor OS, PFS, and RFS. Moreover, the association between pretreatment thrombocytosis and OS is differing according to publication year, country, and sample size, and the association between pretreatment thrombocytosis and PFS could be affected by study quality. The findings of this study indicated pretreatment thrombocytosis was a clinically useful marker to facilitate risk stratification and guide postoperative treatment management.

Numerous systematic review and meta-analysis have already evaluated the role of pretreatment thrombocytosis on prognosis in patients with cancer at various sites. They point out pretreatment thrombocytosis was associated with poor survival for gastric cancer [46, 47], colorectal cancer [48–53], hepatocellular carcinoma [54–56], renal cell carcinoma [57, 58], and endometrial carcinoma [59]. Moreover, a previous meta-analysis illustrated the prognostic value of pretreatment thrombocytosis in patients with gynecologic malignancies, in which patients with thrombocytosis at diagnosis had an increased risk of mortality and patients with gynecologic malignancies had a worse prognosis [60]. The study included only 7 studies that recruited patients with cervical cancer and stratified analysis was not conducted. Another important study found pretreatment thrombocytosis to be an independent prognosis factor of OS and RFS in patients with cervical cancer, whereas it was not associated with PFS [61]. However, several important studies were not included in that study. Moreover, stratified analyses of PFS and RFS were not conducted. Therefore, the current meta-analysis was conducted to identify any new additional information regarding the prognostic role of pretreatment thrombocytosis for patients with cervical cancer.

The summary result of this study found that pretreatment thrombocytosis was associated with a poor OS. Most of the included studies reported similar or non-significant trends for OS and several included studies reported inconsistent results. Wang et al. did not observe a significant association between thrombocytosis before neoadjuvant chemotherapy and OS in patients with early-stage cervical cancer [41]. Li et al. found that thrombocytosis before treatment was associated with an increased risk of mortality, although this association was not statistically significant in Cox regression analysis [36]. Nakamura et al. reported pretreatment thrombocytosis to be associated with improved OS, which was not consistent with the results of previous studies [38]. The potential explanations for this include differences in patient characteristics, treatment strategies, and platelet count cutoff values [29]. Moreover, tumors may induce platelet activation and aggregation in the vasculature, which could cause the expression of angiogenesis regulatory factors [62].

In the present study, pretreatment thrombocytosis was associated with a poor PFS in patients with cervical cancer and only two of the included studies reported consistent results. Koulis et al. indicated that pre-treatment and on-treatment anemia were correlated with worse survival. Moreover, an elevated platelet count was associated with poor OS in patients with various stages [34]. Kozasa et al. reported pretreatment thrombocytosis and elevated platelet-lymphocyte ratio to be independent factors in patients with cervical cancer, and the prognostic role of platelet counts was more sensitive than that of the platelet-lymphocyte ratio [35]. The potential explanation for this finding may be that tumor treatment could promote thrombopoiesis and stimulate cytokines or growth factors, their receptors, or their downstream effectors, which could affect the therapeutic effects in patients with cervical cancer.

The summary results indicated that pretreatment thrombocytosis was correlated with poor RFS in patients with cervical cancer and two of the included studies reported the same conclusions. Zhao et al. included 220 early-stage cervical cancer patients, reporting that the presence of thrombocytosis before treatment was associated with an increased risk of recurrence [44]. Zheng et al. indicated an improved predictive performance with combined platelet count and FIGO, as well as additional risk stratification for operable cervical cancer patients [45]. One possible reason for this significant association could be interaction effects between thrombocytosis and tumor burden. Moreover, platelets might promote tumor vascular growth and platelet receptors and ligands could mediate tumor cell-platelet binding, which could change the biological behavior of the tumors [63, 64].

Subgroup analyses indicated that the prognostic role of pretreatment thrombocytosis on survival outcomes might be affected by the publication year, country, sample size, and study quality. The reason for this observation include (1) treatment strategies have developed rapidly, which could affect the disease prognosis; (2) disease diagnosis and incidence differ between Eastern and Western countries, and disease stage is significantly associated with disease prognosis; (3) sample size was correlated with the weight from the overall analysis and affected the 95% CI of the effect estimate; and (4) study quality was significantly correlated with the evidence level which could have affected the reliability of the pooled results.

This study has several limitations: (1) all of the included studies were retrospective designs, which might induce potential confounders; (2) most of the studies provided crude results for the prognostic role of pretreatment thrombocytosis in patients with cervical cancer; (3) the studies included a wide range of patient characteristics and the heterogeneity among them was not fully interpreted, and further prospective study should be conducted to verify the findings of this study and evaluate the dose-response curve for the association between platelet count before treatment and the prognosis of cervical cancer; (4) the cutoff value and definition of thrombocytosis were differing among included studies, which could affect the prognosis of cervical cancer; and (5) publication bias was inevitable due to the analysis based on published studies and the unavailability of unpublished data.

## Conclusion

In conclusion, the pooled results of this study indicated that thrombocytosis before treatment was associated with a poor prognosis in patients with cervical cancer. The poor prognosis of thrombocytosis before treatment for OS was observed mainly in studies published before 2010, in Western countries, and in large sample sizes. Moreover, the prognostic role of pretreatment thrombocytosis on PFS might differ according to study quality. Further large prospective studies are needed to verify these results and stratified analyses based on patient characteristics should be conducted.

## Additional files

Additional file 1: **Figure S1.** Sensitivity analysis for OS. **Figure S2.** Sensitivity analysis for PFS. **Figure S3.** Sensitivity analysis for RFS. (DOCX 438 kb)

Additional file 2: **Figure S1.** Funnel plot for OS. **Figure S2.** Funnel plot for PFS. **Figure S3.** Funnel plot for RFS. (DOCX 348 kb)

## Data Availability

All data generated or analyzed during this study are included in this published article and its supplementary information files.

## Abbreviations

Cis: Confidence intervals
HRs: Hazard ratios
OS: Overall survival
PFS: Progression-free survival
RFS: Recurrence-free survival
NOS: Newcastle-Ottawa Scale
