# Supplementary material for: Prognostic role of pretreatment thrombocytosis on survival in patients with cervical cancer: a systematic review and meta-analysis
Source: World J Surg Oncol. 2019 Aug 2;17:132. doi: 10.1186/s12957-019-1676-7 (PMC6676533; doi:10.1186/s12957-019-1676-7)
Supplement: Supplementary file 1 — Figure S1. Sensitivity analysis for OS. Figure S2. Sensitivity analysis for PFS. Figure S3. Sensitivity analysis for RFS. (DOCX 438 kb) [file 12957_2019_1676_MOESM1_ESM.docx]

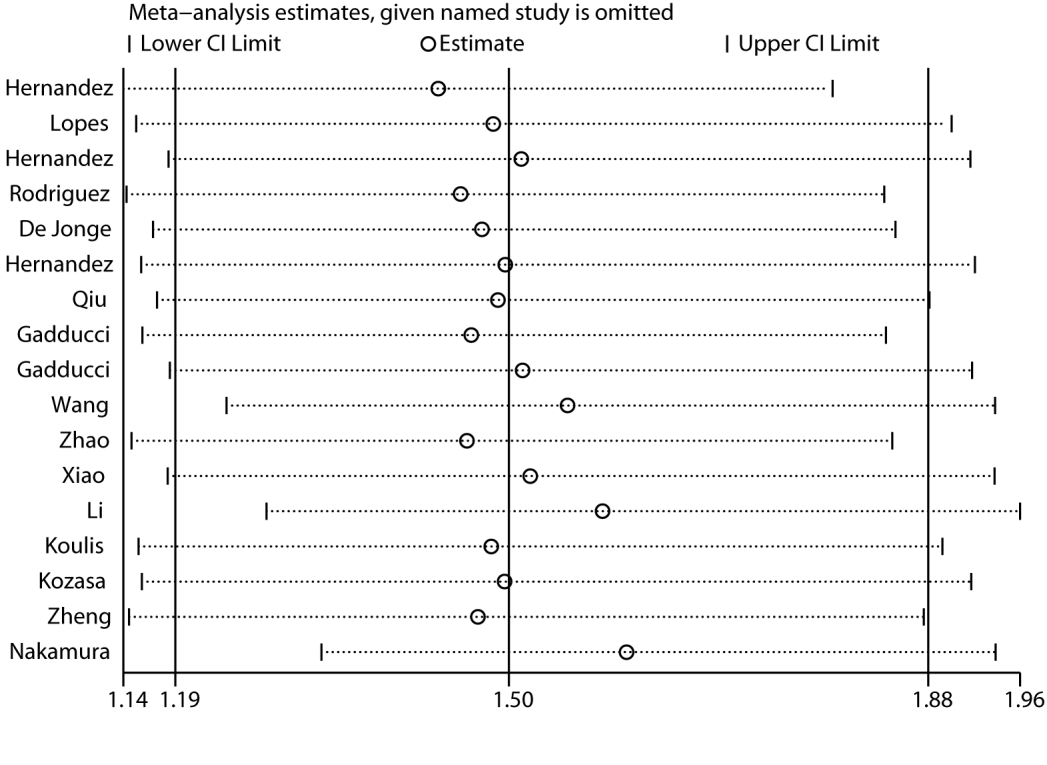


Figure S1. Sensitivity analysis for OS


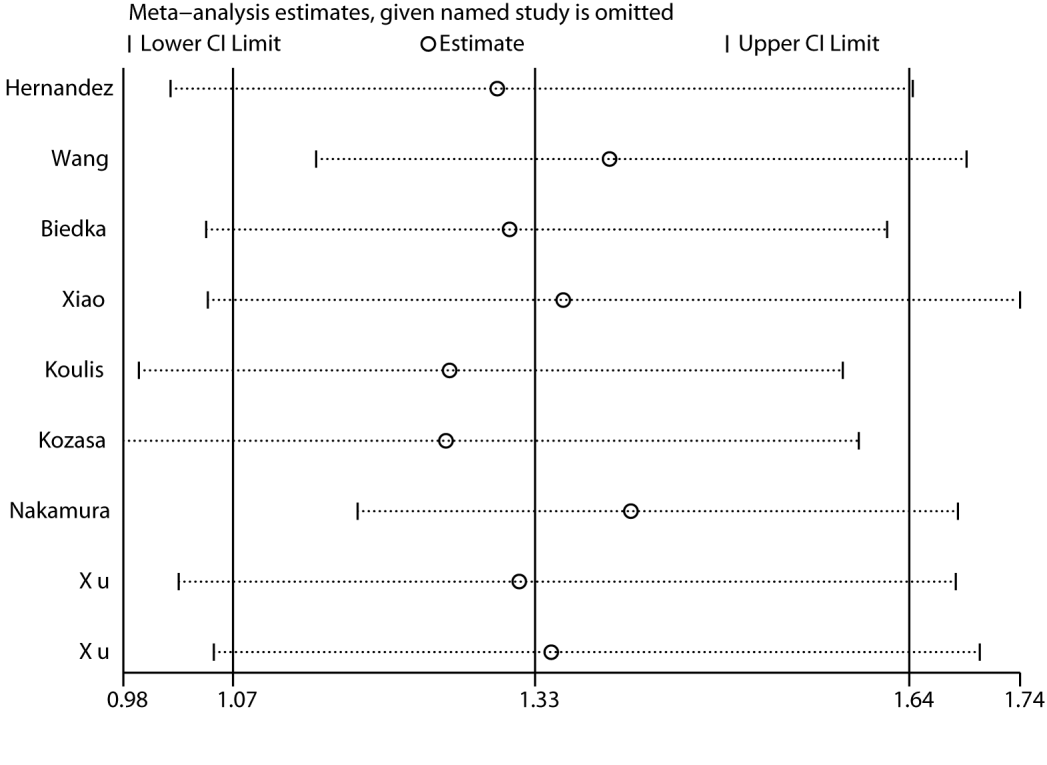


Figure S2. Sensitivity analysis for PFS


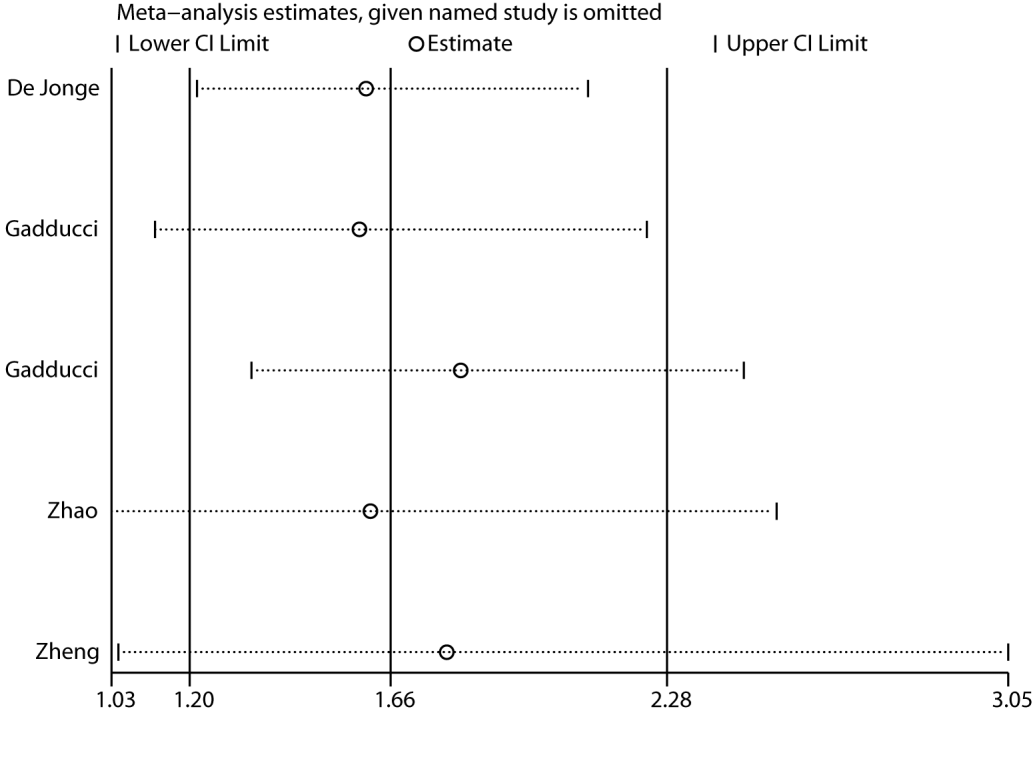


Figure S3. Sensitivity analysis for RFS
