# Supplementary figures and images for: Prognostic role of pretreatment thrombocytosis on survival in patients with cervical cancer: a systematic review and meta-analysis
Source: World J Surg Oncol. 2019 Aug 2;17:132. doi: 10.1186/s12957-019-1676-7 (PMC6676533; doi:10.1186/s12957-019-1676-7)

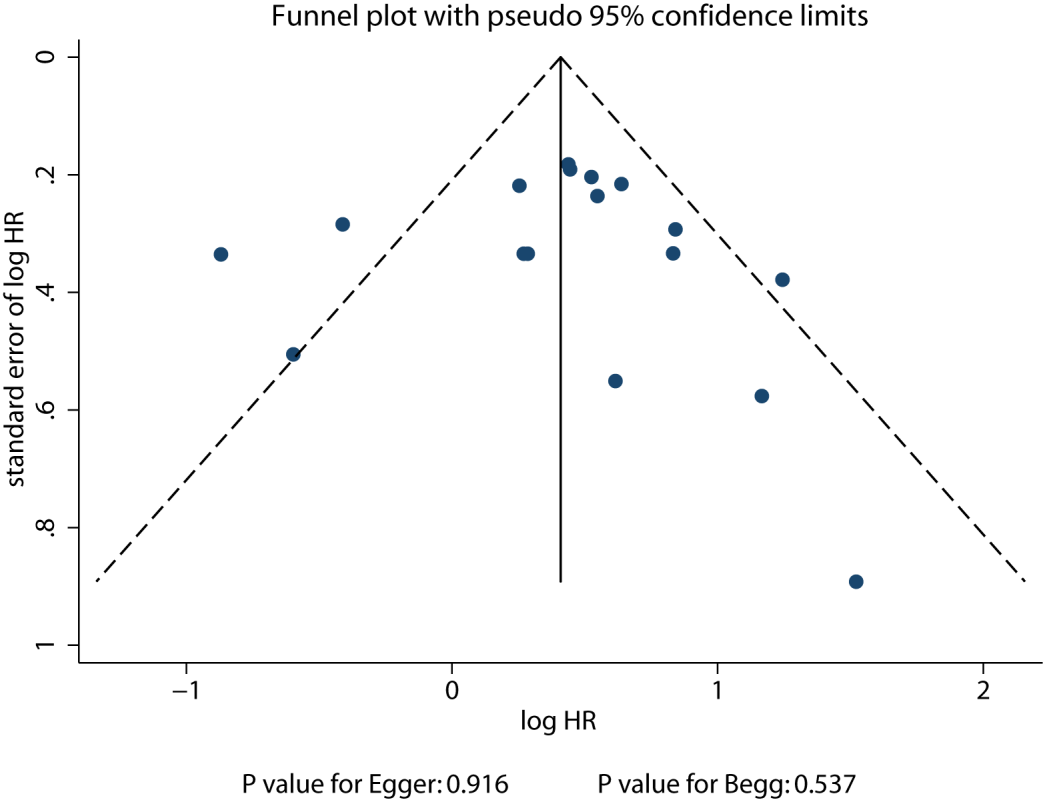


Figure S1. Funnel plot for OS


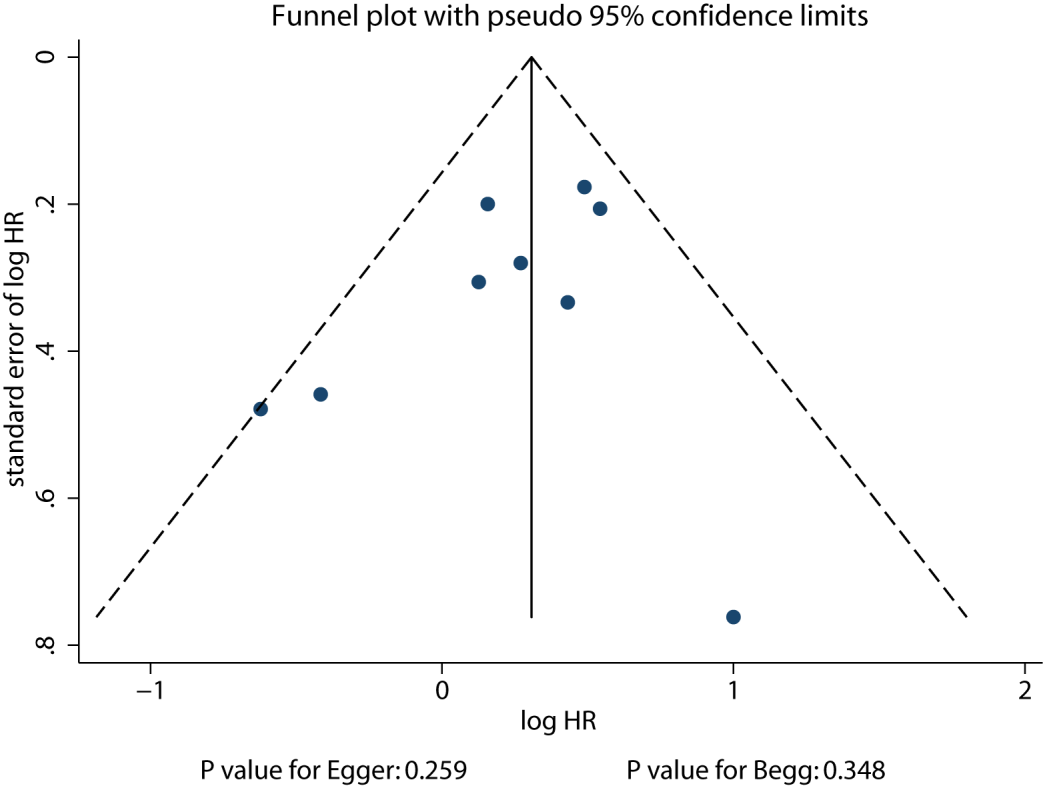


Figure S2. Funnel plot for PFS


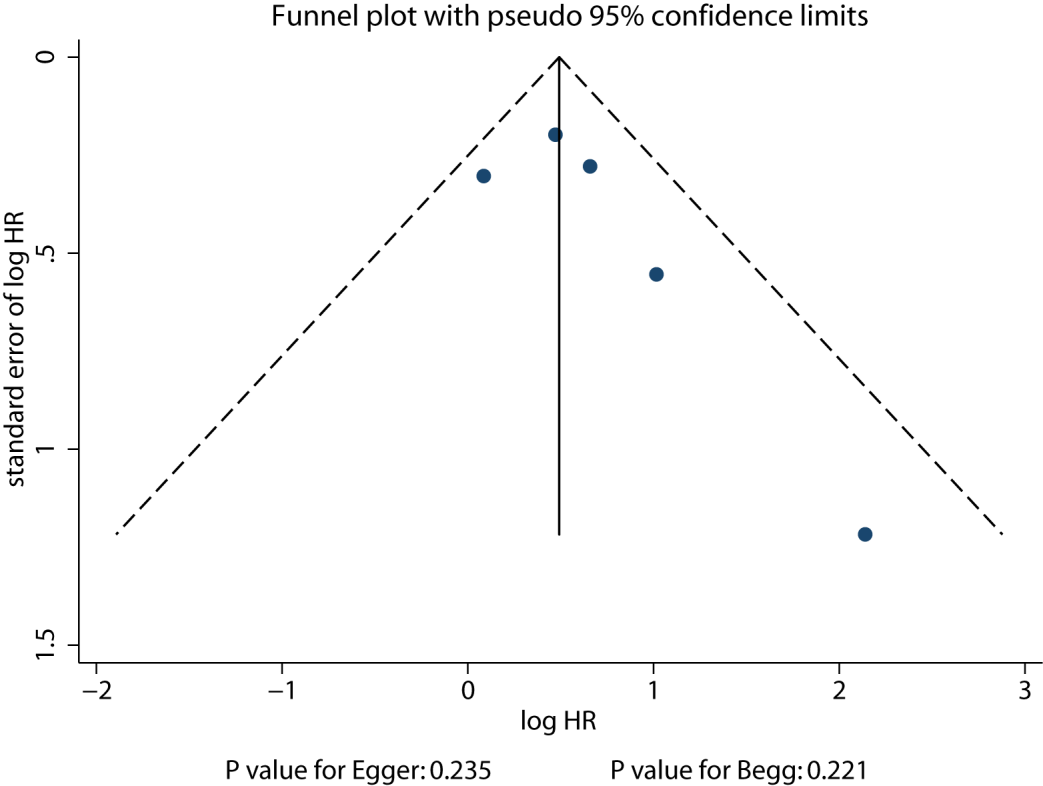


Figure S3. Funnel plot for RFS

Supplement: Supplementary file 2 — Figure S1. Funnel plot for OS. Figure S2. Funnel plot for PFS. Figure S3. Funnel plot for RFS. (DOCX 348 kb) [file 12957_2019_1676_MOESM2_ESM.docx]
